# Factors associated with asthma attack recurrence in Ecuadorian children: longitudinal study of potential impact of the COVID-19 pandemic lockdown

**DOI:** 10.1136/bmjresp-2024-002509

**Published:** 2025-11-27

**Authors:** Santiago Mena-Bucheli, Diana Morillo, Martha Chico, Angelica Ochoa-Aviles, Claudia Rodas-Espinoza, Karen Arteaga, Augusto Maldonado, Alejandro Rodriguez, Camila A Figueiredo, Alvaro A Cruz, Natalia Romero-Sandoval, Irina Chis Ster, Philip J Cooper

**Affiliations:** 1 Escuela de Medicina, Universidad Internacional del Ecuador, Quito, Ecuador; 2 Department of Biosciences, Universidad de Cuenca, Cuenca, Ecuador; 3 Facultad de Medicina, Universidad de Azuay, Cuenca, Ecuador; 4 Emergency Department, Hospital Verdi Cevallos Balda, Portoviejo, Ecuador; 5 Escuela de Medicina, Universidad San Francsico de Quito, Quito, Ecuador; 6 Instituto de Ciencias da Saude, Universidade Federal da Bahia, Salvador, Brazil; 7 Nucleo de Excelencia en Asma, Universidade Federal da Bahia, Salvador, Brazil; 8 Grup's de Recerca d' Amèrica i Àfrica Llatines-GRAAL, Barcelona, Spain; 9 School of Health and Medical Sciences, City St George’s University of London, London, UK

**Keywords:** Asthma Epidemiology, Paediatric asthma

## Abstract

**Background:**

The COVID-19 pandemic caused a major disruption in access to and use of health resources and facilities. There are limited longitudinal data from low-resource settings on the impact of pandemic mitigation strategies and medication use on asthma attacks in children.

**Methods:**

We did a longitudinal study of risk factors for asthma attack recurrence among children aged 5–17 years presenting with an attack to emergency rooms in public hospitals in Ecuador. Children were followed for at least 12 months by monthly telemonitoring. Cox regression models for multiple recurrences were used to identify potential risk factors.

**Results:**

213 asthmatic children were recruited from May 2019 to March 2020 when recruitment was interrupted by a COVID-19 lockdown: 97% were followed for at least 12 months (median 419 days, IQRs 393–421 days). In multivariable analysis, the lockdown effect (adjusted HR 0.35, 95% CI 0.22 to 0.56, p<0.001) and use of inhaled corticosteroids (adjusted HR 0.64, 95% CI 0.43 to 0.93, p=0.020) were strongly protective against recurrence while short-acting β2 agonist use was associated with increased recurrence, particularly among children with a previous asthma diagnosis (interaction p=0.033). Other risk factors were household mould (adjusted HR 1.42, 95% CI 1.03 to 1.95, p=0.031) and number of prerecruitment emergency room visits (adjusted HR 1.05, 95% CI 1.00 to 1.11, p=0.040).

**Conclusion:**

Our data show in a population of asthmatic children from marginalised urban neighbourhoods in Ecuador, that use of inhaled corticosteroids was protective against asthma attack recurrence as were mitigation strategies implemented during the COVID-19 pandemic to reduce transmission of respiratory viruses.

**WHAT IS ALREADY KNOWN ON THIS TOPIC**

- The COVID-19 pandemic had a major impact on access to and use of health resources for chronic diseases such as asthma, particularly in overstretched public health systems in low and middle-income countries (LMICs). There are limited longitudinal data from LMICs on how the pandemic affected asthma attacks in children and adolescents as well as effects of other risk factors such as medication use.

**WHAT THIS STUDY ADDS**

- Mitigation strategies implemented to reduce transmission of SARS-CoV-2 were associated with a reduction in asthma attack recurrence, while use of inhaled corticosteroids appeared to be protective also. In contrast, the use (or likely overuse) of β2 agonists was associated with increased recurrence, particularly among those with a previous doctor’s diagnosis.

**HOW THIS STUDY MIGHT AFFECT RESEARCH, PRACTICE OR POLICY**

- Our findings, from a real-world setting in which asthmatic children and adolescents from low-income neighbourhoods in three Ecuadorian cities were studied, support the use of inhaled corticosteroids in preventing asthma attacks and indicate that strategies to reduce transmission of respiratory viral infections such as the use of masks during periods of intense transmission may prevent attacks.

## Introduction

 Asthma is the most common chronic disease of childhood and is estimated to affect over 350 million people of all ages worldwide.^1^ Epidemiological studies using standardised methodology have shown that the prevalence of childhood asthma in some Latin American cities is as high as reported in high-prevalence populations in high-income countries (HICs).^2^ There is evidence for temporal trends of increasing prevalence in many populations living in low and middle-income countries (LMICs).^2^

In many LMIC settings, asthma is an underdiagnosed and undertreated disease because of limited access to spirometry, specialised care and appropriate treatment.^2^ A combination of inadequate treatment access accompanied by poor adherence^3^ contributes to poor disease control and risk of attacks (or exacerbations), especially in resource-limited settings—under such circumstances asthmatics may rely on emergency services for symptom management,^4^ resulting in unnecessary economic costs and disease burden to health services.^5 6^

Many asthma attacks are preventable,^7^ and identifying modifiable factors associated with risk of attacks will likely reduce associated morbidity and economic costs to health systems. Asthma attacks are associated with anxiety in patients and their families,^8^ high economic costs to health systems and families^9^ as well as loss of lung function and risk of death.^10^ Several previous studies have investigated factors associated with recurrence of asthma attacks among children attending emergency services. In these studies, factors identified as important included a history of previous emergency room attendance for asthma attacks, younger age,^11^,^13^ African ethnicity^14 15^ and low socioeconomic status.^11 16^ Most studies have been done in HICs, with few studies in LMICs.^11 15 16^

We did a prospective study to identify factors associated with recurrence of asthma attacks in children and adolescents attending emergency care in hospitals serving low-resource populations in three Ecuadorian cities. The emergence of the COVID-19 pandemic during the study allowed us to evaluate the potential impact on asthma attacks (and associated risk factors) of mitigation strategies implemented to reduce SARS-COV-2 transmission.

## Methods

### Study design, setting and population

We did a prospective multicentre study in children/adolescents in three Ecuadorian cities (Quito, Cuenca and Portoviejo) as described.^17^ The cities, Quito (population 2.8 million) and Cuenca (0.5 million), are in the Andean highlands at 2850 m and 2560 m altitude, respectively, with average annual temperature of ~16°C, while Portoviejo (0.28 million) is in the central coastal region at sea level with an average temperature of 25°C. Ecuador is an upper middle-income country with a per capita income of US$6080 in 2019. The public health system in Ecuador provides universal health coverage, free at the point of delivery, and is divided into different care levels from primary to tertiary. Children and adolescents attending the emergency department at three tertiary care public hospitals in the three cities (Hospital General Docente de Calderón, Quito; Hospital Vicente Corral Moscoso, Cuenca; and Hospital Regional Verdi Cevallos, Portoviejo—all three hospitals were selected based on convenience) with acute asthma attacks were recruited. Inclusion criteria were age 5–17 years, having a bronchodilator-responsive acute asthma attack while attending the emergency room, living within 12 km distance of the relevant hospital and parental informed written consent and minor assent (for children aged 8 years or older). Exclusions were those with other chronic conditions, who lived more than 12 km from the hospital, or for whom parental informed written consent and minor assent (where applicable) were not obtained. Recruitment was done between May 2019 and March 2020 and had to be suspended on 16th March 2020 following the implementation of a lockdown nationally due to the COVID-19 pandemic. Among eligible patients, an appointment was scheduled at a hospital consulting room within 10–14 days of the attack for recruitment. A total of 213 children and adolescents were recruited, and follow-up was planned for a period of 12 months and last follow-up evaluation was completed in June 2021.

### Study procedures

Standardised procedures were performed by trained teams (physician and nurse) based at each hospital and included at recruitment: (1) baseline questionnaire for information on risk factors, and history of asthma, including core ISAAC asthma symptom questions,^18^ and treatment and management as described previously^11^ and (2) FeNO measured in parts per billion using NObreath (Bedfont Scientific, UK). Monthly follow-ups were done by the same teams using standardised telemonitoring procedures that included a questionnaire to collect data on recurrence of symptoms, unscheduled health facility visits and treatments. The final evaluation at 12 months (or where possible after 12 months) was face-to-face where possible. Medications (inhaled salbutamol and/or fluticasone) were provided free of charge when prescribed by a hospital physician and there was no medication in the hospital pharmacy. The study team ensured adequate inhaler technique during the recruitment evaluation. Study personnel did not interfere with treatment indications of hospital staff.

### Statistical analysis

For analysis, a recurrence of an asthma attack was defined as an episode of acute wheezing during follow-up requiring an unscheduled visit to a health professional (including ER visits) that was controlled by short-acting β2 agonist (SABA) treatment. This definition allowed for changes in health-seeking behaviours during the COVID-19 pandemic and fear of contagion in hospitals.^19^ Originally, we estimated that a sample size of 400 participants would be needed to provide 80% power based on a 50% recurrence during follow-up^11^ and worst-case scenario, which required 16 events for each explanatory variable hypothesised to be 10 (ie, 160 events), taking into account ~20% attrition.^17^ The first recurrence was studied using a Cox proportional hazards model for interval-censored event data to evaluate associations between factors hypothesised to be potential determinants for recurrence. A multivariate outcome defined by multiple recurrences during follow-up was investigated using the Anderson-Gill model for ordered multiple failure events.^20^ In this setting, all failure types are indistinguishable, the clock starts again after the attack and the hazard is interpreted as probability of having an attack at time t *given that the participant has not had any since the last one* (since becoming again at risk if there are no previous attacks); namely, the clock gets reset to zero immediately after each attack. In the context of the former approach, the hazard is interpreted as the probability of having an attack within an interval conditioned on surviving an attack until the lower limit of a specific time interval. The interpretation of results is somewhat similar and uses HRs to measure associations between attacks and hypothesised variables. Both approaches accommodate for multiple records per subject and monthly-varying covariates such as medication use or COVID-19 pandemic lockdown effect, but they are not directly comparable; they use different datasets and different estimation procedures. However, the results were expected to be consistent. To understand the determinants of monthly asthma medications and identify relevant confounders for associations between attack recurrences and medication use, we used generalised estimating equations for longitudinal binary outcomes defined as monthly use of SABA or inhaled corticosteroids (ICS). Finally, a multivariable model for multiple attacks associated with a participant was constructed using complete data and including all potential confounders that were retained using a backward elimination process to obtain the most parsimonious model. This final model was used to derive survival plots for multiple events. Sensitivity analyses for missing data were done, and estimates compared for consistency with the final model in complete data analysis setting. P values less than 0.05 were considered statistically significant. All analyses were done using Stata V.18 (StataCorp, College Station, Texas).

### Ethical considerations

Ethical approval was obtained from the Hospital General Docente de Calderon (CEISH-HGDC 2019–001) and St George’s Research Ethics Committee (Ref 2019.013). Informed written consent to participate was obtained from a parent or guardian, and minor assent for children aged 8 years or older.

### Patient and public involvement statement

Patients and/or the public were not involved in the design, or conduct, or reporting or dissemination plans of this research.

## Results

### Participant characteristics

Of 481 asthmatic children and adolescents evaluated in emergency rooms from May 2019 to March 2020, 230 were eligible and invited to participate of whom 213 were recruited. Of these, 196 (92.0%) completed 12 months of follow-up (eight participants withdrew from the study and contact was lost with 9) (online supplemental figure 1). Of those recruited, 100 (46.9%) had at least one attack recurrence during follow-up. Median follow-up time of the 213 participants was 419 days (IQR 393 to 421 days) and median time to first recurrence of an attack was 90 days (IQR 60–239). The median number of recurrent events was 1 (range 1–5 events). Estimated survival curves for time to first recurrence of an asthma attack and for multiple attacks are shown in online supplemental figure 2.

Individual, socioeconomic and household characteristics, and personal and family history of allergic diseases are shown in table 1. Mean age of participants was 8.6 years (SD 2.9), 51.6% were female and 97.7% were of mestizo ethnicity. Proportions of subjects recruited in the three cities were Quito (24.8%), Cuenca (46.0%) and Portoviejo (29.1%). Household location was 77.1% urban/suburban and 12.7% of households received conditional cash transfers. Recent rhinitis symptoms (76.5%) and flexural dermatitis (27.5%) were frequent among participants. Almost all (96.7%) had follow-up during the period of lockdowns and movement restrictions relating to COVID-19 that started on 16 March 2020. The asthma characteristics of participants are provided in table 2. The majority (71.5%) had a doctor diagnosis of asthma. During the 12 months before the asthma attack at recruitment: most (93.7%) reported wheezing symptoms and a mean of 2.7 (SD 2.5) attacks during this period; 57.7% reported ER attendance with asthma symptoms, and 12.7% were hospitalised; and 88.3% reported no use of ICS in the previous year (only 8.5% used ICS regularly).

**Table 1:** Risk factors (individual, socioeconomic and household, and personal and family history of allergic diseases) for asthma attack recurrence among 213 participants

Variable	Category/summary	All	Attack recurrence		HR	95% CI	P value
			No	Yes			
**Subjects**	n	213	100	113			
Individual characteristics							
Age	Mean/SD	8.6/2.9	8.8/3.0	8.5/2.7	0.99	0.93 to 1.05	0.740
Sex	Male	103 (48.4%)	46 (46%)	57 (50.4%)	1		
	Female	110 (51.6%)	54 (54%)	56 (49.6%)	0.91	0.65 to 1.26	0.560
Ethnicity	Mestizo	201 (97.7%)	95 (95%)	106 (93.8%)	1		
	Non-Mestizo	12 (2.3%)	5 (5%)	7 (6.2%)	0.65	0.40 to 1.05	0.080
BMI	Mean/SD	18.6/4.3	19.0/4.6	18.6/4.3	0.99	0.95 to 1.03	0.476
Breastfeeding	Never	10 (4.7%)	3 (3.0%)	7 (6.3%)			
	0–6 months	27 (12.8%)	10 (10.1%)	17 (15.2%)	1.55	0.83 to 2.88	0.166
	>6 months	174 (82.5%)	86 (86.9%)	88 (78.5%)	1.07	0.66 to 1.72	0.794
Socioeconomic and household							
Site	Quito	53 (24.8%)	17 (17%)	36 (31.9%)	1		
	Cuenca	98 (46.0%)	45 (45%)	53 (46.9%)	0.86	0.59 to 1.24	0.410
	Portoviejo	62 (29.1%)	38 (38%)	24 (21.2%)	**0.52**	**0.32 to 0.85**	**0.009**
Maternal education level	None/Basic	39 (21.1%)	22 (25.0%)	17 (17.5%)	1		
	Secondary/high	146 (78.9%)	66 (75.0%)	80 (82.5%)	1.17	0.72 to 1.88	0.723
Dog in house	No	154 (72.3%)	72 (72.0%)	82 (72.6%)			
	Yes	59 (27.7%)	28 (28.0%)	31 (27.4%)	0.91	0.62 to 1.33	0.615
Cat in house	No	186 (87.3%)	84 (84.0%)	102 (90.3%)	0.65	0.37 to 1.15	0.143
	Yes	27 (12.7%)	16 (16.0%)	11 (9.7%)	**2.16**	**1.81 to 2.56**	**<0.001**
Second-hand smoke	No	194 (92.4%)	91 (91.9%)	103 (92.8%)			
	Yes	16 (7.6%)	8 (8.1%)	8 (7.2%)	1.13	0.58 to 2.17	0.722
Mould in house	No	183 (85.9%)	91 (91.0%)	92 (81.4%)			
	Yes	30 (14.1%)	9 (9.0%)	21 (18.6%)	**1.54**	**1.04 to 2.27**	**0.031**
House location	Rural	47 (22.1%)	19 (19%)	28 (24.8%)	1		
	Urban	166 (77.1%)	81 (81%)	85 (75.2%)	1.00	0.70 to 1.42	0.990
Household overcrowding	<3 per room	154 (72.3%)	75 (75.0%)	79 (69.9%)	1		
	≥3 per room	59 (27.7%)	25 (25.0%)	34 (30.1%)	0.93	0.67 to 1.31	0.693
House location	No	104 (51.5%)	54 (56.3%)	50 (47.2%)	1		
>50 m main street	Yes	98 (48.5%)	42 (43.7%)	56 (52.8%)	1.07	0.76 to 1.50	0.700
Receipt of conditional cash transfer (bono)	No	185 (87.3%)	90 (90.1%)	95 (84.1%)	1		
	Yes	27 (12.7%)	9 (8.9%)	18 (15.9%)	**1.54**	**1.02 to 2.32**	**0.038**
Personal and family history							
Bronchiolitis history before 2 years	No	150 (72.5%)	68 (69.4%)	82 (75.2%)	1		
	Yes	57 (27.5%)	30 (30.6%)	27 (24.8%)	0.89	0.59 to 1.36	0.604
Rhinitis last 12 m	No	47 (23.5%)	21 (23.3%)	26 (23.6%)	1		
	Yes	153 (76.5%)	69 (76.7%)	84 (76.4%)	**1.46**	**1.01 to 2.13**	**0.047**
Eczema last 12 m	No	145 (72.5%)	74 (77.9%)	71 (67.6%)	1		
	Yes	55 (27.5%)	21 (22.1%)	34 (32.4%)	1.29	0.90 to 1.84	0.164
Maternal asthma	No	180 (85.7%)	87 (88.8%)	93 (83.0%)	1		
	Yes	30 (14.3%)	11 (11.2%)	19 (17.0%)	1.29	0.82 to 2.02	0.270
Maternal rhinitis	No	150 (71.4%)	77 (79.4%)	73 (64.6%)	1		
	Yes	60 (28.6%)	20 (20.6%)	40 (35.4%)	**1.41**	**1.01 to 1.97**	**0.046**
Paternal asthma	No	177 (87.6%)	86 (92.5%)	91 (83.5%)	1		
	Yes	25 (12.4%)	7 (7.5%)	18 (16.5%)	**1.75**	**1.15 to 2.67**	**0.009**
Paternal rhinitis	No	146 (72.6%)	73 (77.7%)	73 (68.2%)	1		
	Yes	55 (27.2%)	21 (22.3%)	34 (31.8%)	**1.56**	**1.10 to 2.21**	**0.013**
Pandemic effect (longitudinal) with follow-up	Before	7 (3.3%)	5 (5.0%)	2 (1.8%)	1		
After 16 March	After	206 (96.7%)	95 (95.0%)	111 (98.2%)	**0.41**	**0.26 to 0.65**	**<0.001**

Variables are stratified by the presence of any asthma attack recurrence while associations are estimated (HR for one or more asthma attacks (ie, multiple events) during follow-up. Missing: breastfeeding (n=2); maternal educational level (28); second-hand smoke (3); house location >50m street (11); receipt of bono (1); bronchiolitis (6); rhinitis last 12 m (13); flexural dermatitis last 12 m (13); maternal asthma (3); maternal rhinitis (3); paternal asthma (11); paternal rhinitis (12).

Statistically significant P values (P<0.05) are shown in bold.

**Table 2:** Risk factors (asthma history and medications) for asthma attack recurrence among 213 participants

Variable	Category/summary	All	Attack recurrence		HR	P value	95% CI
			No	Yes			
Subjects	n	213	100	113			
Asthma characteristics							
Wheezing last 12 months	No	13 (6.3%)	7 (7.1%)	6 (5.5%)	1		
	Yes	195 (93.7%)	92 (92.9%)	103 (94.5%)	0.95	0.881	0.46 to 1.96
Number attacks last 12 m	Mean/SD	2.7/2.5	2.3/2.0	3.0/2.8	**1.07**	**0.019**	**1.01 to 1.14**
Exercise-induced wheeze last 12 m	No	49 (26.2%)	24 (28.2%)	25 (24.5%)			
	Yes	138 (73.8%)	61 (71.8%)	77 (75.5%)	1.14	0.473	0.79 to 1.65
FeNO (log scale)	Mean/SD	3.1/1.5	2.8/1.6	3.4/1.3	**1.21**	**0.005**	**1.06 to 1.38**
Medical attention history							
Previous doctor diagnosis asthma	No	57 (28.1%)	31 (32.6%)	26 (24.1%)			
	Yes	146 (71.9%)	64 (67.4%)	82 (75.9%)	**1.52**	**0.032**	**1.04 to 2.24**
ER visits last 12 m	Mean (SD)	1.9/2.4	1.5/1.7	2.3/2.7	**1.08**	**0.009**	**1.02 to 1.14**
Hospitalisation/ICU last 12 m	No	186 (87.3%)	92 (92%)	94 (83.2%)	1		
	Yes	27 (12.7%)	8 (8%)	19 (16.8%)	1.24	0.264	0.85 to 1.81
Time since the last ER visit	Never	176 (82.6%)	79 (79%)	97 (85.8%)	1		
in the last 12 months	<30 days	13 (6.1%)	8 (8%)	5 (4.4%)	0.62	0.273	0.264 to 1.46
	30–150 days	15 (7.0%)	8 (8%)	7 (6.2%)	0.89	0.727	0.47 to 1.70
	>150 days	9 (4.2%)	5 (5%)	4 (3.5%)	0.92	0.890	0.30 to 2.89
OCS use (≥3 days) over last 12 m	<3 days	173 (81.2%)	82 (82%)	91 (80.5%)	1		
	≥3 days	40 (18.8%)	18 (18%)	22 (19.5%)	1.29	0.349	0.76 to 2.18
B2 agonist use over last 12 m	None	172 (80.7%)	85 (85%)	87 (77%)			
	Wheezing only	33 (15.5%)	13 (13%)	20 (18%)	1.20	0.417	0.77 to 1.87
	Regularly	8 (3.8%)	2 (2%)	6 (5%)	1.63	0.109	0.90 to 2.97
ICS use over last 12 m	None	188 (88.3%)	91 (91%)	97 (86%)	1		
	Wheezing only	7 (3.3%)	2 (2%)	5 (4.4%)	0.92	0.788	0.51 to 1.67
	Regularly	18 (8.5%)	7 (7%)	11 (9.7%)	1.29	0.378	0.74 to 2.24
Medication use during follow-up (longitudinal)							
SABA (time-varying)	No	105 (49%)	82 (82%)	23 (20%)	1		
	Yes	108 (51%)	18 (18%)	90 (80%)	**2.13**	**<0.001**	**1.61 to 2.81**
ICS (time-varying)	No	114 (54%)	59 (59%)	55 (49%)	1		
	Yes	99 (46%)	41 (41%)	58 (51%)	0.76	0.158	0.52 to 1.11

Variables are stratified by presence of any asthma attack recurrence while associations are estimated (HR for one or more asthma attacks [ie, multiple events]) during follow-up. Medication use during follow-up with proportions showing medication use during follow-up and HRs estimate effect on recurrence risk of medication use in previous month as time-varying variable. Missing: wheeze last 12 m (n=5) and exercise-induced wheeze last 12 m (26).

Statistically significant P values (P<0.05) are shown in bold.

ER, emergency room; FeNO, fractional exhaled nitric oxide; ICS, inhaled corticosteroids; ICU, intensive care unit; OCS, oral corticosteroids; SABA, short-acting β2 agonist.

### Univariate associations between risk factors and asthma attack recurrence

Associations of individual, sociodemographic, household and familial factors with having one or more attacks (ie, complete follow-up considered allowing multiple potential events) are provided in table 1, while associations of asthma history and medications are shown in table 2. The analysis for the first attack as an interval-censored event is provided in online supplemental tables 1 and 2. Factors associated with having one or more attacks in univariate analyses were study site (Portoviejo vs Quito, HR 0.52, 95% CI 0.32 to 0.85, p=0.009); having a household cat (HR 2.16, 95% CI 1.81 to 2.56, p<0.001); having mould in the house (HR 1.54, 95% CI 1.04 to 2.27, p=0.031); receipt of conditional cash transfer by the child’s family—an indicator of severe poverty (HR 1.54, 95% CI 1.02 to 2.32, p=0.038); having rhinitis during the previous 12 months (HR 1.46, 95% CI 1.01 to 2.13, p=0.047); maternal (HR 1.41, 95% CI 1.01 to 1.97, p=0.046) and paternal (HR 1.56, 95% CI 1.15 to 2.67, p=0.009) rhinitis; paternal asthma (HR 1.56, 95% CI 1.10 to 2.21, p=0.013); follow-up during the pandemic period (HR 0.41, 95% CI 0.26 to 0.65, p<0.001); number of asthma attacks during previous 12 months (HR 1.07, 95% CI 1.01 to 1.14, p=0.019); a previous asthma diagnosis (HR 1.52, 95% CI 1.04 to 2.24, p=0.032); number of unplanned asthma consultations over previous 12 months (HR 1.05, 95% CI 1.00 to 1.10, p=0.033); and baseline levels of FeNO (1.21-fold change per log, 95% CI 1.06 to 1.38, p=0.005). During follow-up, SABA use (taken in the previous month) was strongly associated with at least one attack (HR 2.13, 95% CI 1.61 to 2.91, p<0.001) while there was a non-significant inverse trend with ICS use (HR 0.76, 95% CI 0.52 to 1.11, p=0.158). Univariate determinants of use during follow-up of SABA, ICS and oral corticosteroids are shown in online supplemental tables 3 and 4. Numerous variables were associated with increased use of SABA and ICS, such as non-mestizo ethnicity, having other allergic diseases or parents with allergy, and the number of asthma attacks or unplanned medical consultations in the previous year. Differences in medication use, with significantly more SABA and less ICS, were seen among children whose family received conditional cash transfers (an indicator of severe poverty), among those with follow-up during the COVID-19 pandemic, and those recruited in Quito.

### Multivariable associations between risk factors and asthma attack recurrence

**Figure 1:**
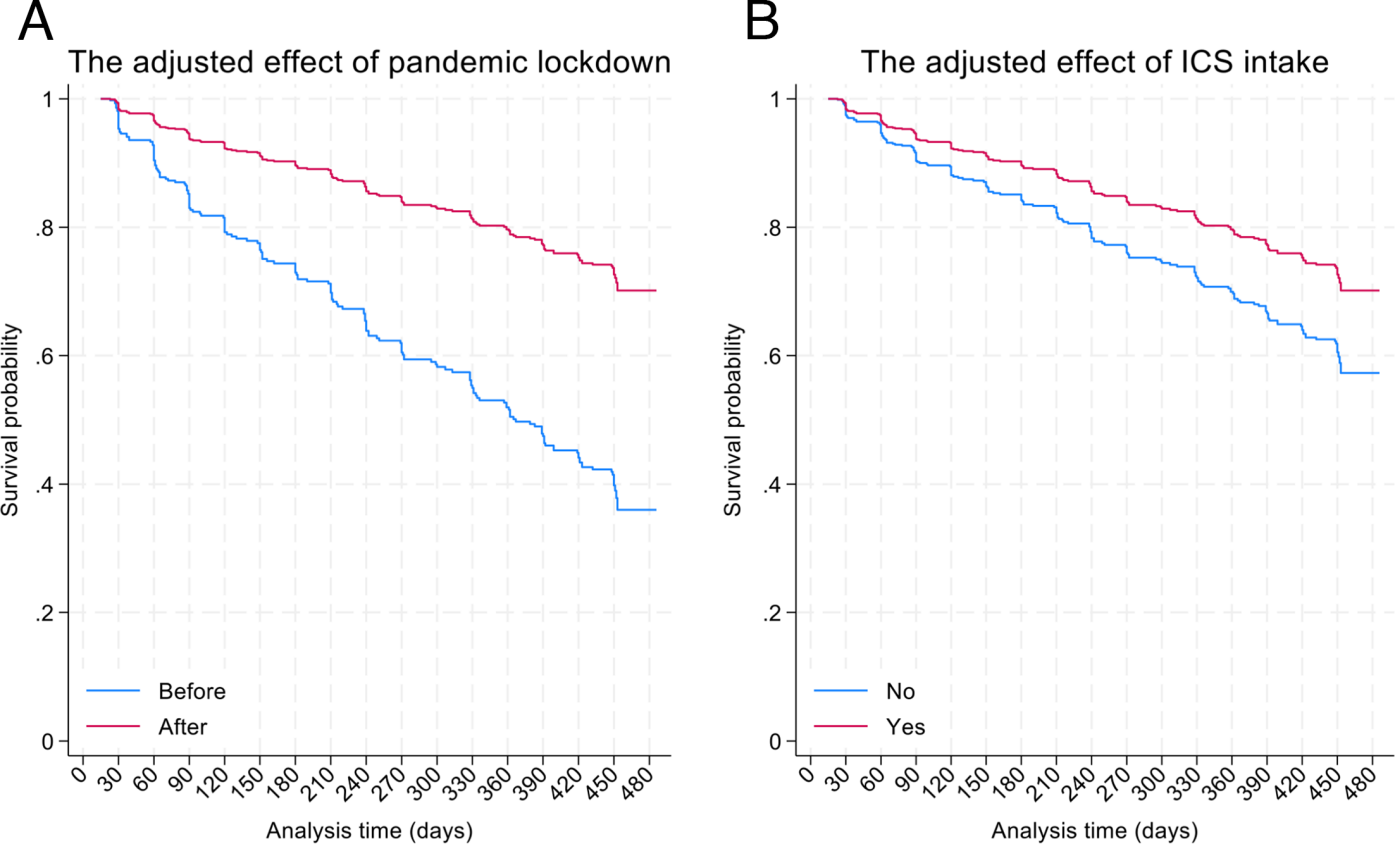
Effects of COVID-19 pandemic lockdown (from 16 March 2020) (**A**) and other subsequent mitigation strategies and of use of inhaled corticosteroids (**B**) on estimated survival curve for multiple recurrent attacks. Estimated curves were fitted using the most parsimonious model to a longitudinal binary outcome defined by multiple attacks. For the pandemic effect, other variables were set to: number of unplanned medical consultations, 1 (the median); ‘No’ for mould in the house, ‘No’ to SABA use, ‘No’ for doctor diagnosis; and assuming ‘Yes’ for ICS use. For the effect of ICS intake, other variables are set to: number of unplanned medical consultations, 1 (the median); ‘No’ for mould in the house, ‘No’ to SABA use, ‘No’ for doctor diagnosis; and ‘During’ for pandemic. ICS, inhaled corticosteroids; SABA, short-acting b2 agonists.

**Figure 2:**
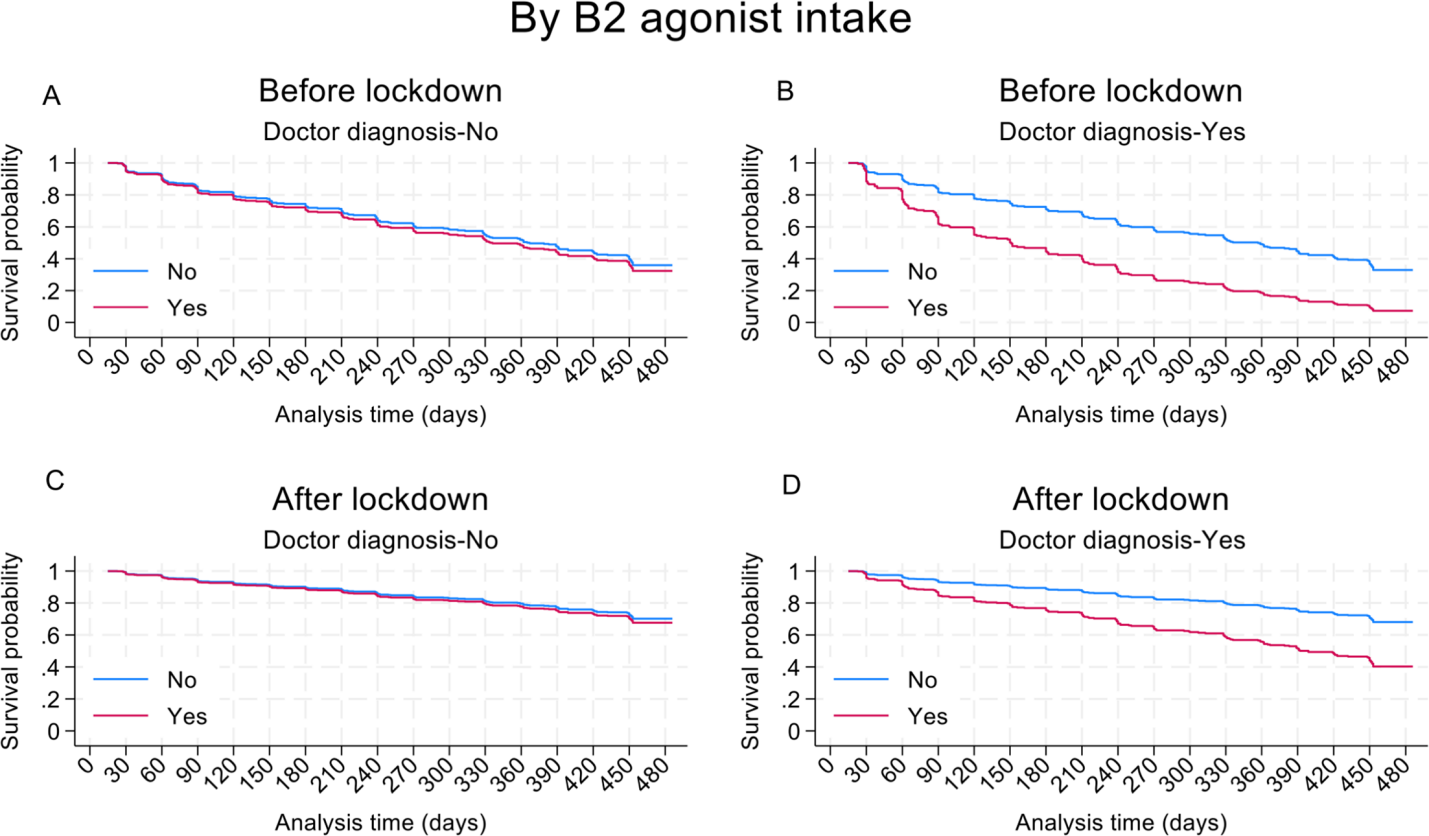
Effect of use of short-acting beta2 agonists (SABA) on estimated survival curve for multiple recurrent attacks stratified by pandemic (before and during) and doctor diagnosis. Estimated curves were fit using the most parsimonious model. Other variables are set to: number of unplanned medical consultations, 1 (the median); ‘No’ for mould in the house; ‘No’ to SABA use and asthma diagnosis for A and B, and ‘Yes’ for C and D. at ‘Yes’ for ICS intake and ‘No’ to B2 agonist intake and at ‘No’ for asthma diagnosed by a physician for (**A, B**) and to ‘Yes’ for (**C, D**).

**Figure 3:**
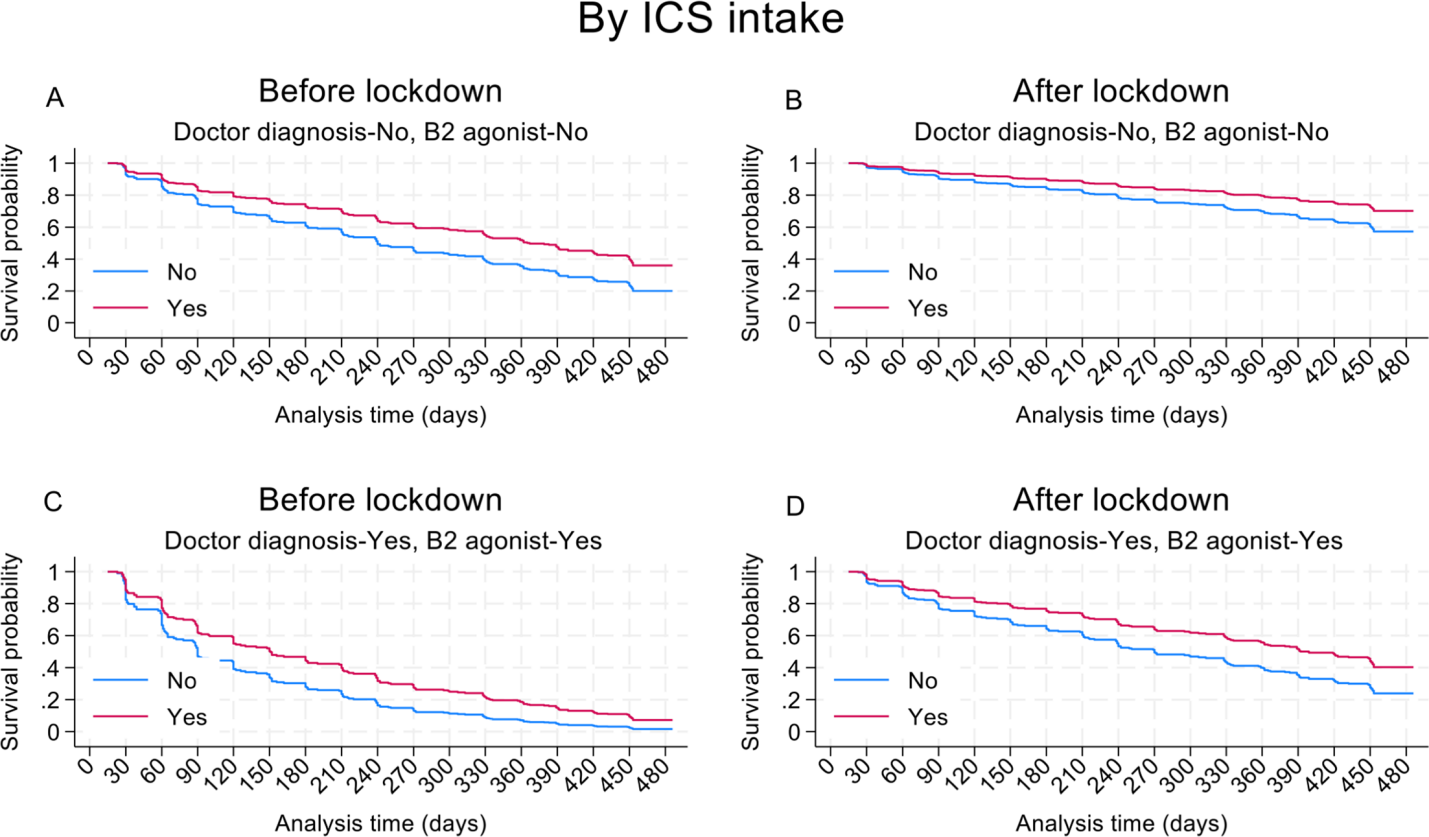
Effect of use of inhaled corticosteroids (ICS) on estimated survival curve for multiple recurrent attacks showing effect of ICS use before and after pandemic lockdown, and by β2 agonist and doctor diagnosis of asthma. Estimated curves were fitted using the most parsimonious model to a longitudinal binary outcome defined by multiple attacks. Other variables are set to: number of unplanned medical consultations, 1 (the median); ‘No’ for mould in the house; ‘Yes’ to ICS use; and “No” to asthma diagnosis for (A & B), and “Yes” for (C & D).

Results for the most parsimonious multivariable model of factors associated with at least one recurrent attack during follow-up are provided in table 3. Having follow-up during the pandemic (adjusted OR 0.35, 95% CI 0.22 to 0.56, p<0.001) (figure 1A) and use of ICS (adjusted HR 0.64, 95% CI 0.43 to 0.93, p=0.020) (figure 1B) were protective against recurrence. Factors significantly associated with recurrence of attacks were mould on the walls of the home (adjusted HR 1.42, 95% CI 1.03 to 1.95, p=0.031), and number of ER visits for asthma symptoms in 12 months before recruitment (per visit, adjusted HR 1.05, 95% CI 1.00 to 1.11, p=0.040). There was an interaction between a previous doctor’s diagnosis of asthma and SABA use during follow-up such that recurrence was more likely among children having a doctor diagnosis and taking SABA (interaction p=0.033), an effect that did not appear to be affected by the pandemic lockdown (figure 2). The protective effect of ICS use against recurrent attacks was present irrespective of the pandemic lockdown, SABA use and doctor diagnosis (figure 3). Results of sensitivity analyses for missing data showed comparable findings (online supplemental table 5).

**Table 3:** Multivariable associations between risk factors and asthma attack recurrence

Variable	Category	HR	95% CI	P value
Mould on walls of home	No	1		
	Yes	1.419	1.03 to 1.95	0.031
ER visits last 12 m	Count	1.052	1.00 to 1.11	0.040
Pandemic effect (longitudinal) with follow-up after 16 March 2020	Before	1		
	After	0.347	0.22 to 0.56	<0.001
ICS use in previous month (time-varying)	No	1		
	Yes	0.636	0.43 to 0.93	0.020
Previous doctor diagnosis of asthma	No	1		
	Yes	1.085	0.69 to 1.72	0.728
SABA use in previous month (time-varying)	No	1		
	Yes	1.103	0.58 to 2.10	0.767
Interaction for Doctor Diagnosis×SABA Use		1		
		2.146	1.07 to 4.32	0.033

Shown are associations (HR) of risk factors with multiple attacks during follow-up derived from the most parsimonious model following a complete data analysis. A strict lockdown was imposed nationally from 16 March 2020.

A strict lockdown was imposed nationally from 16 March 2020.

ER, emergency room; ICS, inhaled corticosteroid; SABA, short-acting b2 agonist.

## Discussion

The prevalence of childhood asthma has increased in many LMICs over recent years where asthma attacks are a growing burden to health systems.^2^ Asthma attacks are associated with significant morbidity and economic costs, and there are limited data on determinants of attacks in children and adolescents from low-resource LMIC settings.^16^ In the present study, we identified factors associated with recurrence of asthma attacks among children and adolescents living in marginal neighbourhoods in a range of climatic settings in highland Andean and Coastal cities in Ecuador. We recruited asthmatics presenting with an asthma attack to the ER in hospitals in three cities and followed them up for at least 12 months. The study was interrupted by the COVID-19 pandemic with a lockdown implemented in March 2020 that interrupted study recruitment but provided the opportunity to study the effect on attack recurrence of strict restrictions on movement and social interactions. Our data showed that the implementation of mitigation strategies to limit transmission of SARS-CoV-2 (ie, social distancing, mask wearing and travel restrictions) including a 3-month lockdown and 2 years of virtual schooling, were associated with a marked reduction in likelihood of recurrence while, independently, use of ICS was protective also. Factors associated with increased hazard included SABA use, a previous doctor diagnosis, the number of ER visits for asthma in the year prior to recruitment and household mould. Our data provide real-world evidence from a low-resource setting to support the use of ICS in asthmatic children to reduce risk of asthma attacks, and indirect evidence for the importance of respiratory viral infections in contributing to asthma attacks in such populations.

This longitudinal study done in ERs at public hospitals serving low-resource populations in three Ecuadorian cities allowed us to study the real-world situation of asthma care in these settings in contrast to the highly controlled and selected populations of clinical trials. The study team did not interfere with patient management although SABA and/or ICS were provided free when these medications were not available through the hospital pharmacy. The study was done in two temperate high-altitude cities (Quito and Cuenca) and one tropical low-altitude city (Portoviejo), making our findings broadly generalisable to similar urban settings and populations in Ecuador and other countries in Andean Latin America with similar healthcare systems. Study power will have been reduced by the recruitment of a smaller than expected sample size (recruitment was interrupted by the COVID-19 lockdowns) and a reduction in expected number of events: the pandemic had a major impact on health-seeking behaviours while mitigation strategies to control SARS-CoV-2 transmission resulted in marked declines in the circulation of respiratory viruses that cause most asthma attacks in children.^21^ However, our study did have sufficient power to detect relatively large effects. Follow-up was high, with 93% of participants completing 12 months or more of follow-up, thus limiting any potential selection bias. Losses to follow-up during the pandemic were limited by a shift to telemonitoring for all follow-up activities. Although the same outcome definition and instruments were used, changes in how data were collected (ie, the shift from face-to-face to telemonitoring for a minority of follow-ups) could potentially have resulted in misclassification. Because access to mobile phones was universal in this population, the shift to follow-up solely by telemonitoring likely had a negligible or limited effect on ascertainment of study outcomes. Monthly follow-ups likely reduced missing events, and confounding was minimised by collecting data on a wide variety of known potential confounders. Although we were able to evaluate longitudinally the effects of medication use on recurrence of attacks, we were not able to assess adherence or inhaler technique during follow-up.

There are few previous prospective studies in low-resource settings in LMICs investigating risk factors for asthma attacks in children and adolescents. Among previous studies, a cohort of asthmatics aged 5–15 years using similar methodology to our study and done in the Ecuadorian coastal city of Esmeraldas identified younger age, previous asthma diagnosis, number of courses of corticosteroids in the previous year and eczema as factors associated with recurrence of attacks.^11^ Asthma diagnosis was the only factor retained as significant in multivariable analysis. Possible explanations for lack of consistency between these two studies^11^ include differences in study populations (eg, ethnicity—very few Afro-Ecuadorians, the predominant ethnicity in the Esmeraldas study, were recruited in the present study), access to healthcare (<1% of the children in Esmeraldas were receiving ICS on recruitment compared with 8.5% in this study) and effects of the pandemic in reducing power to detect smaller effects.

There is extensive evidence from high-income settings that ICS, even when taken as needed as combined therapy in mild asthmatics, is highly effective in preventing asthma attacks in children and adults.^22^ A previous longitudinal analysis of trial data from children with mild-to-moderate asthma in the US showed that ICS reduced risk of attacks by around 40%.^7^ A recent randomised clinical trial among black and Hispanic asthmatic adults in the USA showed that provision of ICS, in addition to usual care, reduced risk of attacks by about 15%.^23^ There are more limited non-trial real-world data on the effectiveness of ICS in reducing risk of asthma attacks from low-resource settings in LMICs. In this study, done in such a setting, ICS use was highly protective (36% reduction) against attacks. Our study population, largely represented by asthmatics with mild-to-moderate disease based on medication use, should normally be managed adequately through primary care. However, because of limited access to asthma medications, accompanied by poor training in asthma management at this level, a lack of access to specialised care and poor co-ordination of care between different levels of the health system, such asthmatics often present repeatedly to emergency services for symptom control.^24^

In contrast to ICS, SABA use was associated with increased attack recurrences, and the effect was strongest for those with a previous diagnosis, likely reflecting greater access to and use of SABA. Estimates of SABA overuse among asthmatics from HICs are highly variable but can be as high as 40%.^25 26^ A link between SABA overuse for symptom relief and increased hazard of asthma attacks and death has been known since the early 1990s,^27 28^ and SABA use alone is no longer recommended for the treatment of mild disease.^29^ A recent analysis of national data from Sweden showed that SABA overuse (defined as three or more canisters per year) was associated with an increased risk of attacks and deaths.^30^ Furthermore, a study of asthmatics in Canada, where 25% of those with mild disease had SABA overuse, showed that the association between SABA overuse and increased risk of attacks persisted even when stratified by ICS use.^26^

Our study was interrupted on 16 March 2020 by the imposition of a strict national lockdown to reduce SARS-CoV-2 transmission during the COVID-19 pandemic. The combination of the lockdown and interruption of routine activities in the study hospitals that were reassigned to COVID-19 activities resulted in the suspension of both recruitment and face-to-face follow-up. The pandemic caused dramatic changes in access to and use of health facilities by asthmatic children in Ecuador either because services were suspended or because of fear of contagion among patients and their families.^19^ The strict lockdown in March 2020 lasted approximately 2 months and was followed by restrictions on movement and social distancing with mandatory mask-wearing in public places lasting a further 4 months. Furthermore, public schooling at a national level was provided remotely for 2 years following the initial lockdown. These measures had a dramatic effect on the circulation of non-SARS-CoV-2 respiratory viruses that were reduced to very low levels for almost a year after the initial lockdown.^31^ Most (ie, up to 90%) asthma attacks in children have been attributed to respiratory viral infections,^21 32^ particularly rhinovirus but also respiratory syncytial virus (RSV),^21^ which may explain the almost 60% reduction in risk of attack recurrence among those with follow-up following the COVID-19 lockdown. Other studies from HICs have shown dramatic reductions in ER consultations and hospitalisations for paediatric asthma following the implementation of lockdowns during the COVID-19 pandemic.^33^,^35^ The relationship between SARS-CoV-2 infection and asthma attack risk is unclear, although there is some evidence that later virus variants might be associated with increased risk.^36^ Other factors that might have contributed to the pandemic effect in reducing asthma attacks include reduced air pollution^37^ and better treatment adherence.^38^

Our data provide strong support for the widely implemented strategy (Global Initiative for Asthma, GINA) advocating the use of combination inhalers with β2 agonists and ICS in the management of even mild asthma in low-resource LMIC settings to minimise morbidity, mortality and economic costs associated with asthma attacks. There is still a limited evidence base in LMICs for the use of combination inhalers (or prescription of separate SABA and ICS inhalers) which, although often included in national lists of essential drugs by public health systems, are frequently not prioritised for procurement. Furthermore, our findings provide further evidence from such settings for the increased risk of asthma attacks associated with SABA-only therapy and SABA overuse.^26 29 30^ Because RSV, and to a lesser extent rhinovirus circulation, is seasonal even in tropical coastal regions of Ecuador,^39 40^ the wearing of masks by asthmatic children with a history of severe attacks, might be an effective method to reduce risk of subsequent attacks and associated morbidity. Now that mask-wearing has become more normalised following the COVID-19 pandemic, future studies could explore the potential benefits of mask-wearing by children and adolescents in schools during seasonal periods of more intense circulation of respiratory viruses (ie, during the months of January through March representing the rainy season in Ecuador) as a strategy to reduce risk of attacks.

The COVID-19 pandemic had a major effect in reducing the risk of asthma attack recurrence in asthmatic children and adolescents living in low-income neighbourhoods in three Ecuadorian cities. The use of ICS independently protected against attacks while SABA use increased risk, particularly among those with a doctor diagnosis of asthma. There is a need for improved education of physicians about the use of ICS as a first-line treatment for asthma and risks of SABA overuse. Public health systems in LMICs should prioritise the procurement of combination inhalers to minimise morbidity and deaths caused by asthma attacks and the associated economic costs of poorly controlled disease. Future studies could evaluate the usefulness of mask-wearing in children and adolescents during periods of more intense seasonal transmission of respiratory viruses as a strategy to reduce risk of asthma attacks.

## Supplementary material

10.1136/bmjresp-2024-002509 online supplemental figure 1

10.1136/bmjresp-2024-002509 online supplemental figure 2

10.1136/bmjresp-2024-002509 online supplemental table 1

## Data Availability

Data are available upon reasonable request.
