# Supplementary figures and images for: Factors associated with asthma attack recurrence in Ecuadorian children: longitudinal study of potential impact of the COVID-19 pandemic lockdown
Source: BMJ Open Respir Res. 2025 Nov 27;12(1):e002509. doi: 10.1136/bmjresp-2024-002509 (PMC12684217; doi:10.1136/bmjresp-2024-002509)

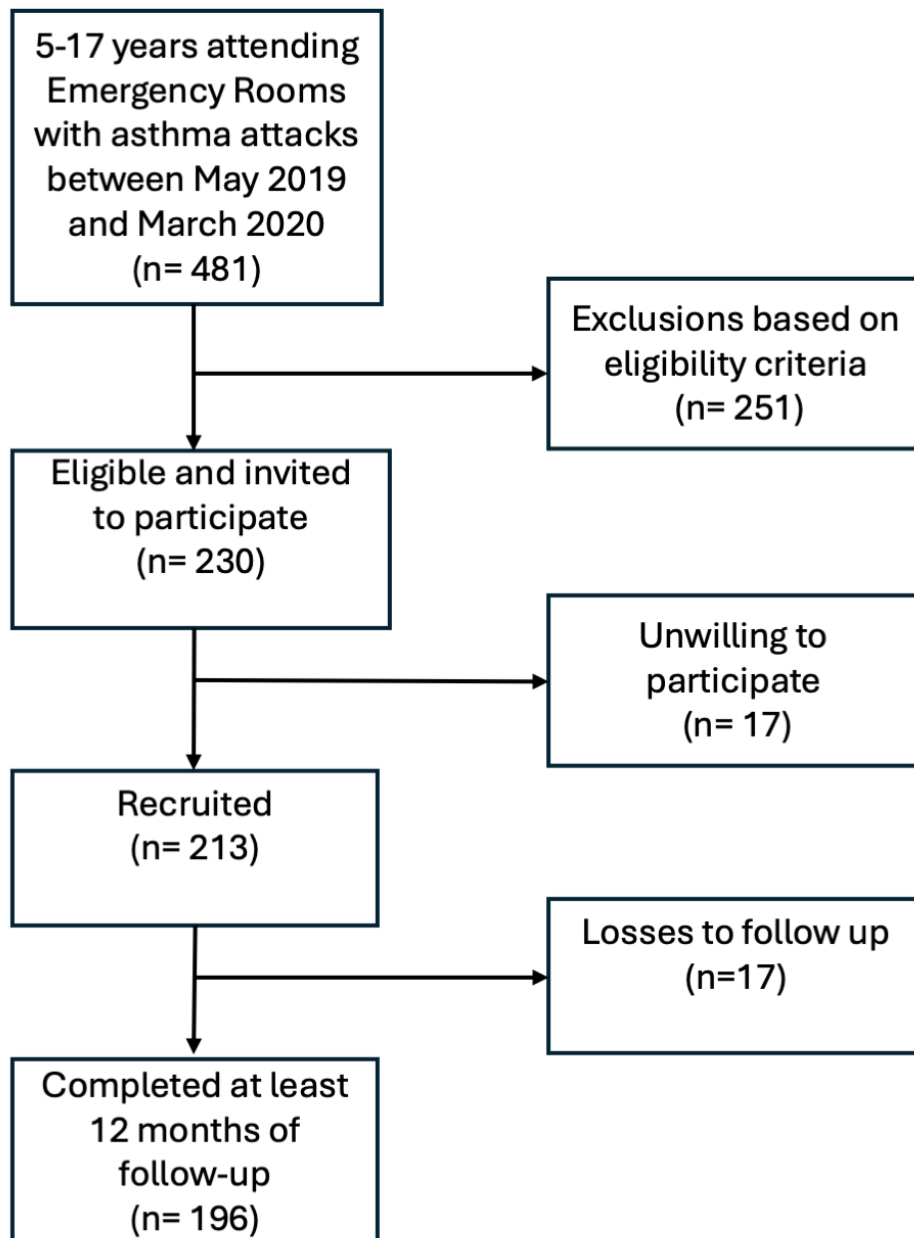

Supplement: online supplemental figure 1 [file bmjresp-12-1-s001.pdf]

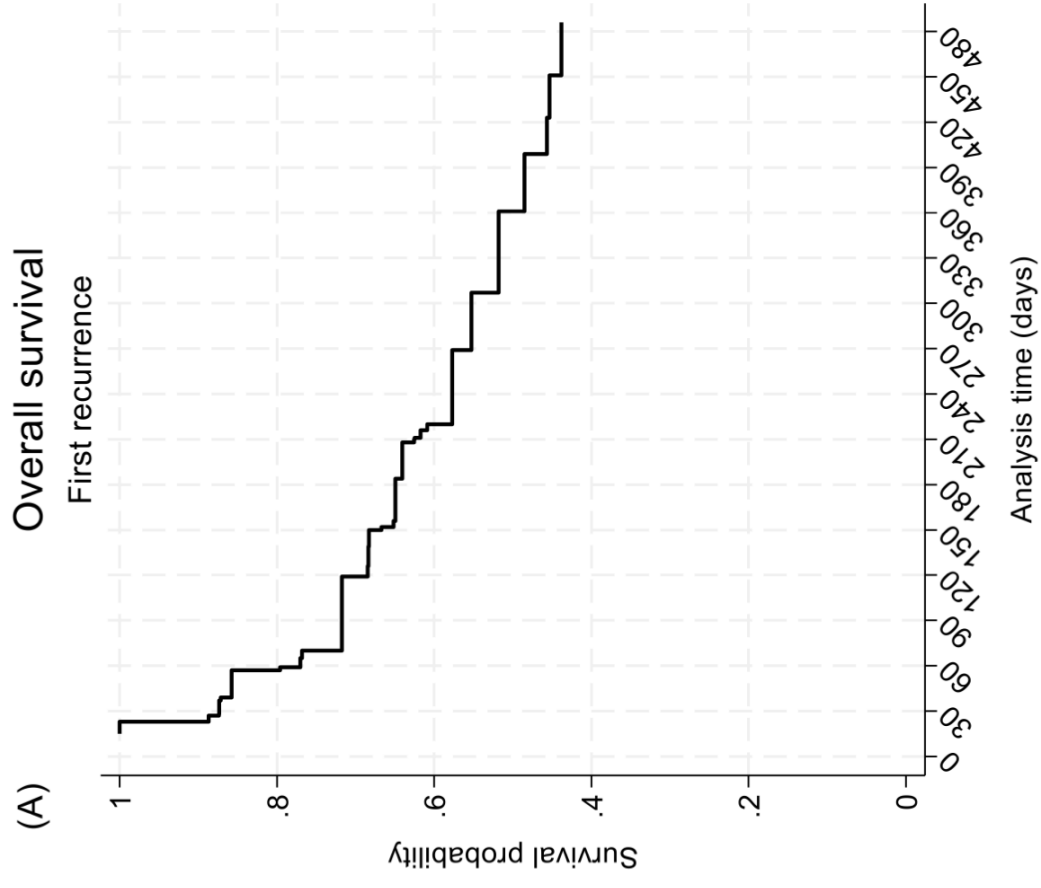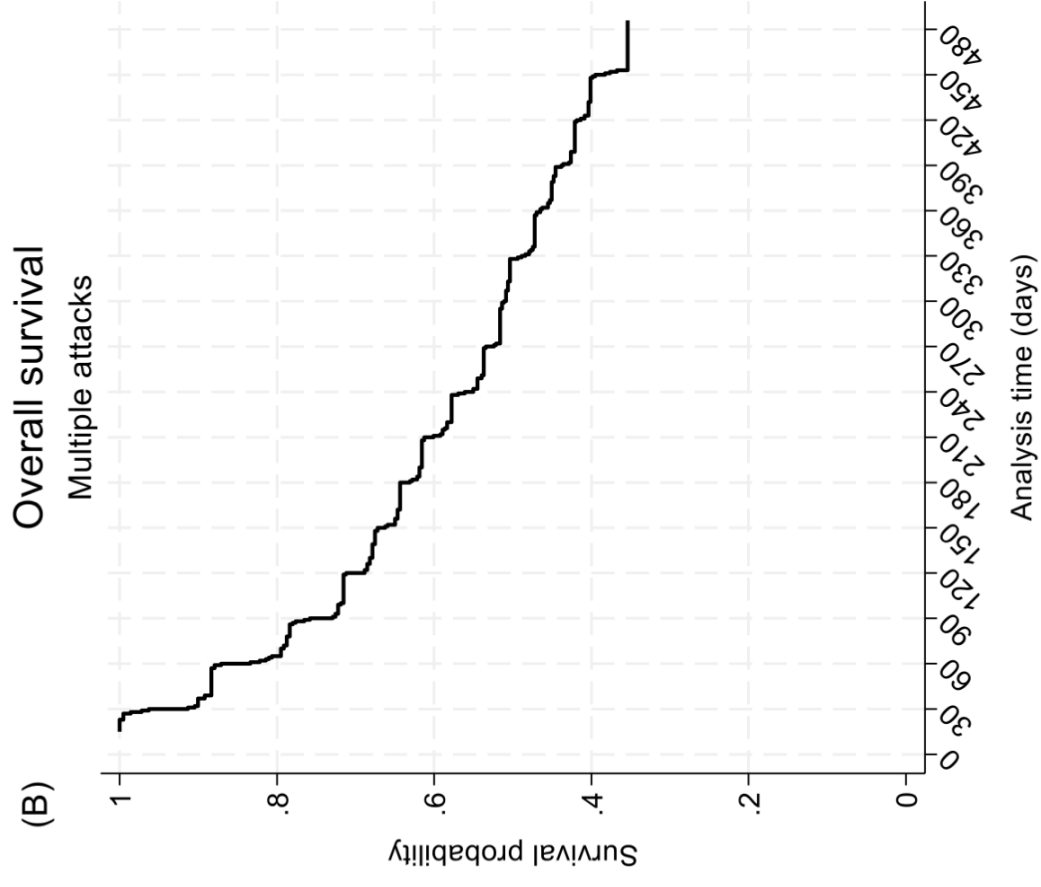

Supplement: online supplemental figure 2 [file bmjresp-12-1-s002.pdf]
