# Supplementary material for: Factors associated with asthma attack recurrence in Ecuadorian children: longitudinal study of potential impact of the COVID-19 pandemic lockdown
Source: BMJ Open Respir Res. 2025 Nov 27;12(1):e002509. doi: 10.1136/bmjresp-2024-002509 (PMC12684217; doi:10.1136/bmjresp-2024-002509)
Supplement: online supplemental table 1 [file bmjresp-12-1-s003.docx]

|  |  |  |  |  | First attack analysis | | | |
| --- | --- | --- | --- | --- | --- | --- | --- | --- |
| Variable | Category/Summary | Attack never | Attack ever | All | Interval censored (113 failures) | | | |
|  | Subsequent | 100(47%) | 113(53%) | 213 | Multiple intervals per participant | | | |
| **Individual characteristics** |  |  |  |  |  |  |  |  |
| Age | Mean/SD | 8.8/3.0 | 8.5/2.7 | 8.6/2.9 | 0.985 | 0.647 | 0.923 | 1.051 |
| Sex | Male | 46(46%) | 57(50.4%) | 103(48.4%) |  |  |  |  |
|  | Female | 54(54%) | 56(49.6%) | 110(51.6%) | 0.893 | 0.549 | 0.617 | 1.292 |
| Ethnicity | Mestizo | 95(95%) | 106(93.8%) | 201(97.7%) | 1 |  |  |  |
|  | Non-Mestizo | 5(5%) | 7(6.2%) | 12(2.3%) | 1.277 | 0.518 | 0.608 | 2.683 |
| BMI | Mean/SD | 19.0/4.6 | 18.6/4.3 | 18.6/4.3 | 0.977 | 0.322 | 0.933 | 1.023 |
| Breastfeeding | Never | 3(3.0%) | 7(6.2%) | 10 (4.7%) | 1 |  |  |  |
|  | 0-6 months | 10(10.0%) | 17(15.0%) | 27(12.7%) | 0.766 | 0.533 | .331 | 1.770 |
|  | >6 months | 86(86.0%) | 88(77.9%) | 174(36.2%) | 0.533 | 0.092 | .257 | 1.108 |
|  | Missing | 1(1.0%) | 1(0.9%) | 2(0.9%) |  |  |  |  |
| FeNO (log scale) | Mean/SD | 3.1/1.5 | 2.8/1.6 | 3.4/1.3 | **1.262** | **0.002** | **1.091** | **1.460** |
| **Socio-economic and household** |  |  |  |  |  |  |  |  |
| Site | Quito | 17(17%) | 36(31.9%) | 53(24.8%) | 1 |  |  |  |
|  | Cuenca | 45(45%) | 53(46.9%) | 98 (46.0%) | 0.719 | 0.106 | 0.481 | 1.073 |
|  | Portoviejo | 38(38%) | 24(21.2%) | 62 (29.1%) | 0.382 | **0.001** | 0.222 | 0.657 |
| Maternal education level | None/Basic | 22(22.0%) | 17(15.0%) | 39(18.3%) | 1 |  |  |  |
|  | Some secondary/hi | 66(66.0%) | 80(70.7%) | 146(68.6%) | 1.314 | 0.285 | .796 | 2.169 |
|  | Missing | 12(12.0%) | 16(14.2%) | 28(13.2%) |  |  |  |  |
| Dog in house | No | 72(72.0%) | 82(72.6%) | 154(72.3%) |  |  |  |  |
|  | Yes | 28(28.0%) | 31(27.4%) | 59 (27.7%) | 0.890 | 0.588 | 0.585 | 1.355 |
| Cat in house | No | 84(84.0%) | 102(90.3%) | 186 (87.3%) |  |  |  |  |
|  | Yes | 16(16.0%) | 11(9.7%) | 27 (12.7%) | 0.607 | 0.128 | 0.319 | 1.154 |
| Second-hand smoke exposure | No | 91(91.0%) | 103(91.2%) | 194(91.1%) |  |  |  |  |
|  | Yes | 8(8.0%) | 8(7.1%) | 16 (7.5%) | 0.868 | 0.707 | 0.417 | 1.810 |
|  | Missing | 1(1.0%) | 2(1.8%) | 3 (1.4%) |  |  |  |  |
| Mould in house | No | 91(91.0%) | 92(81.4%) | 183(85.9%) |  |  |  |  |
|  | Yes | 9(9.0%) | 21(18.6%) | 30(14.1%) | 1.764 | **0.019** | 1.096 | 2.841 |
| House location | Rural | 19(19%) | 28(%) | 47(22.1%) | 1 |  |  |  |
|  | Urban/Suburban | 81(81%) | 85(%) | 166(77.1%) | .818 | 0.382 | .522 | 1.282 |
| Household overcrowding | <3 per room | 75(%) | 79(%) | 154(%) | 1 |  |  |  |
|  | >=3 per room | 25(%) | 34(%) | 59(%) | 1.150 | 0.498 | .768 | 1.721 |
| House location | No | 54(54.0%) | 50(44.3%) | 104 (48.8%) |  |  |  |  |
| >50m main street | Yes | 42(42.0%) | 56(49.6%) | 98 (46.0%) | 1.382 | 0.097 | 0.943 | 2.026 |
|  | Missing | 4(4.0%) | 7(6.2%) | 11(5.2%) |  |  |  |  |
| Receipt of Condition Cash Transfer | No | 90(90.0%) | 95(84.1%) | 185 (86.9%) |  |  |  |  |
| (bono) | Yes | 9(9.0%) | 18(15.9%) | 27(12.7%) | 1.725 | **0.026** | 1.068 | 2.787 |
|  | Missing | 1(1.0%) | 0(0.0%) | 1 (0.5%) |  |  |  |  |
| **Personal and family history** |  |  |  |  |  |  |  |  |
| Bronchiolitis history before 2 years | No | 68(68.0%) | 82(72.6%) | 150(70.4%) |  |  |  |  |
|  | Yes | 30(30.0%) | 27(23.9%) | 57 (26.8%) | 0.818 | 0.354 | 0.535 | 1.251 |
|  | Missing | 2(2.0%) | 4(3.5%) | 6 (2.8%) |  |  |  |  |
| Rhinitis last 12 m | No | 21(21.0%) | 26(23.0%) | 47(22.1%) |  |  |  |  |
|  | Yes | 69(69.0%) | 84(74.3%) | 153(71.8%) | 1.359 | 0.181 | 0.867 | 2.131 |
|  | Missing | 10(10.0%) | 3(2.7%) | 13(6.1%) |  |  |  |  |
| Flexural dermatitis last 12m | No | 74(74.0%) | 71(62.8%) | 145(68.1%) |  |  |  |  |
|  | Yes | 21(21.0%) | 34(30.1%) | 55(25.8%) | 1.423 | 0.084 | 0.954 | 2.123 |
|  | Missing | 5(5.0%) | 8(7.1%) | 13(6.1%) |  |  |  |  |
| Maternal asthma | No | 87(87.0%) | 93(82.3%) | 180 (84.5%) |  |  |  |  |
|  | Yes | 11(11.0%) | 19(16.8%) | 30 (14.1%) | 1.255 | 0.383 | 0.754 | 2.090 |
|  | Missing | 2(2.0%) | 1(0.9%) | 3 (1.4%) |  |  |  |  |
| Maternal rhinitis | No | 77(77.0%) | 73(64.6%) | 150 (70.4%) |  |  |  |  |
|  | Yes | 20(20.0%) | 40(35.4%) | 60 (28.2%) | 1.601 | **0.017** | 1.086 | 2.359 |
|  | Missing | 3(3.0%) | 0(0.0%) | 3 (1.4%) |  |  |  |  |
| Paternal asthma | No | 86(86.0%) | 91(80.5%) | 177(%) |  |  |  |  |
|  | Yes | 7(7.0%) | 18(15.9%) | 25 (%) | 1.709 | **0.024** | 1.075 | 2.717 |
|  | Missing | 7(7.0%) | 4(3.5%) | 11 (%) |  |  |  |  |
| Paternal rhinitis | No | 73(73.0%) | 73(64.6%) | 146(68.5%) |  |  |  |  |
|  | Yes | 21(21.0%) | 34(30.1%) | 55 (25.8%) | 1.402 | 0.104 | 0.933 | 2.107 |
|  | Missing | 6(6.0%) | 6(5.3%) | 12 (5.6%) |  |  |  |  |
| Pandemic effect (longitudinal) | Before | 5(5.0%) | 2(1.8%) | 7(3.3%) | 1 |  |  |  |
| Follow-up after 16^th^ March | After | 95(95.0%) | 111(98.2%) | 206 (96.7%) | 0.386 | **0.003** | 0.205 | 0.725 |

**Supplementary Table 1. Risk factors (individual, socio-economic and household, asthma history and medications) for asthma attack recurrence among 213 participants.** Univariate analyses are shown for the effect of risk factors on the first recurrent attack treated as an interval-censored survival outcome. Missing: breastfeeding (n=2); maternal educational level (28); second-hand smoke (3); house location >50m street (11); receipt of bono (1); bronchiolitis (6); rhinitis last 12 m (13); flexural dermatitis last 12 m (13); maternal asthma (3); maternal rhinitis (3); paternal asthma (11); paternal rhinitis (12). SD – standard deviation; FeNO – fractional exhaled nitric oxide.

**Supplementary Table 2. Risk factors (asthma history and medications) for asthma attack recurrence among 213 participants.** Univariate analyses for the effect of risk factors on first recurrent attack treated as an interval-censored survival outcome. Variables are stratified by presence of any asthma attack recurrence. Medication use during follow-up with proportions showing medication use during follow-up and HRs estimate effect on recurrence risk of medication use in previous month as time-varying variable. Missing: Wheeze last 12 m (n=5) and Exercise-induced wheeze last 12 m (26). SD – standard deviation; ER – emergency room; ICU -intensive care unit; OCS – oral corticosteroids; ICS – inhaled corticosteroids; SABA – short-acting β2 agonist.

|  |  |  |  |  | First attack analysis | | | |
| --- | --- | --- | --- | --- | --- | --- | --- | --- |
| Variable | Category/Summary | Attack never | Attack ever | All | Interval censored (113 failures) | | | |
|  | Subsequent | 100(47%) | 113(53%) | 213 | Multiple intervals per participant | | | |
| **Asthma characteristics** |  |  |  |  | 1877 patient-intervals | | | |
| Wheezing last 12 months | No | 7(7.0%) | 6(5.3%) | 13(6.1%) | 1 |  |  |  |
|  | Yes | 92(92.0%) | 103(91.2%) | 195 (91.6 %) | 1.132 | 0.768 | 0.497 | 2.574 |
|  | Missing | 1(1.0%) | 4(3.5%) | 5(2.4%) |  |  |  |  |
| Number attacks last 12m | Mean/SD | 2.3/2.0 | 3.0/2.8 | 2.7/2.5 | 1.100 | **0.005** | 1.030 | 1.175 |
| Exercise-induced wheeze last 12 m | No | 24(24.0%) | 25(22.1%) | 49(23%) |  |  |  |  |
|  | Yes | 61(61%) | 77(68.1%) | 138(64.8%) | 1.334 | 0.168 | .886 | 2.009 |
|  | Missing | 15(15.0%) | 11(9.7%) | 26(12.2%) |  |  |  |  |
| Previous doctor diagnosis asthma | No | 31(31.0%) | 26(23.0%) | 57(26.8%) |  |  |  |  |
|  | Yes | 64(64.0%) | 82 (72.6%) | 146(68.5%) | 1.263 | 0.288 | 0.821 | 1.945 |
|  | Missing | 5(5.0%) | 5(4.4%) | 10(4.5%) |  |  |  |  |
|  |  |  |  |  |  |  |  |  |
| ER last 12 months | No | 46(46.0%) | 44(38.9%) | 90(42.3%) |  |  |  |  |
|  | Yes | 54(54.0%) | 69(61.1%) | 123(57.7%) | 1.301 | 0.181 | 0.885 | 1.913 |
| ER visits last 12 months | Mean/SD | 1.4/1.8 | 2.3/3.4 | 1.9/2.8 | 1.111 | **0.006** | 1.03 | 1.199 |
| Hospitalization/ICU last 12m | No | 92(92%) | 94(83.2%) | 186(87.3%) | 1 |  |  |  |
|  | Yes | 8(8%) | 19(16.8%) | 27(12.7%) | 1.433 | 0.187 | 0.839 | 2.448 |
| Time since the last ER visit | Never | 79(79%) | 97(85.8%) | 176 (82.6%) | 1 |  |  |  |
| in the last 12 months | <30 days | 8(8%) | 5(4.4%) | 13 (6.1%) | 0.709 | 0.367 | 0.337 | 1.495 |
|  | 30-150 days | 8(8%) | 7(6.2%) | 15 (7.0%) | 0.980 | 0.953 | 0.510 | 1.884 |
|  | >150 days | 5(5%) | 4(3.5%) | 9 (4.2%) | 0.800 | 0.630 | 0.322 | 1.986 |
| OCS use (>=3 days) over last 12 m | <3 days | 82(82%) | 91(80.5%) | 173(81.2%) | 1 |  |  |  |
|  | >=3 days | 18(18%) | 22(19.5%) | 40(18.8%) | 1.096 | 0.691 | 0.696 | 1.726 |
| β 2 agonist use over last 12m | None | 85(85%) | 87(77%) | 172(80.7%) | 1 |  |  |  |
|  | Wheezing only | 13(13%) | 20(18%) | 33(15.5%) | 1.423 | 0.140 | 0.890 | 2.273 |
|  | Regularly | 2(2%) | 6(5%) | 8(3.8%) | 2.154 | **0.047** | 1.010 | 4.594 |
| ICS use over last 12m | None | 91(91%) | 97(86%) | 188(88.3%) | 1 |  |  |  |
|  | Wheezing only | 2(2%) | 5(4.4%) | 7(3.3%) | 1.866 | 0.110 | 0.868 | 4.012 |
|  | Regularly | 7(7%) | 11(9.7%) | 18(8.5%) | 1.266 | 0.452 | 0.685 | 2.338 |
| **Medication use during follow-up (longitudinal)** |  |  |  |  |  |  |  |  |
| SABA (time-varying) | Never vs. | 82(82%) | 23(20%) | 105(49%) |  |  |  |  |
|  | Ever (p<0.001) | 18(18%) | 90(80%) | 108(51%) | 2.079 | **<0.001** | 1.39 | 3.107 |
| ICS (time-varying) | Never vs. | 59(59%) | 55(49%) | 114(54%) |  |  |  |  |
|  | Ever (p=0.144) | 41(41%) | 58(51%) | 99(46%) | 1.406 | 0.086 | .953 | 2.075 |
| OCS (time-varying) | Never vs. | 93(93%) | 98(87%) | 191(90%) |  |  |  |  |
|  | Ever(p=0.148) | 7(7%) | 15(13%) | 22(10%) | 2.432 | **0.032** | 1.077 | 5.491 |

| **Variables** |  | All | β2 agonist | | | | Inhaled corticosteroid | | | | Oral corticosteroid | | | |
| --- | --- | --- | --- | --- | --- | --- | --- | --- | --- | --- | --- | --- | --- | --- |
|  |  |  | OR | p-value | 95%CI - L | 95%CI - H | OR | p-value | 95%CI - L | 95%CI - H | OR | p-value | 95%CI - L | 95%CI - H |
| Follow-up (months) |  |  | 0.991 | 0.123 | 0.979 | 1.003 | 0.980 | **0.001** | 0.969 | 0.991 | 1.002 | 0.914 | 0.971 | 1.034 |
| **Individual characteristics** |  |  |  |  |  |  |  |  |  |  |  |  |  |  |
| Age | Mean/SD | 8.6/2.9 | 1.056 | **<0.001** | 1.038 | 1.075 | 0.976 | **0.004** | 0.959 | 0.992 | 1.014 | 0.558 | 0.969 | 1.060 |
|  | Median (Q1-Q3) | 8 (6-11) |  |  |  |  |  |  |  |  |  |  |  |  |
|  | Min-Max | 5-17 |  |  |  |  |  |  |  |  |  |  |  |  |
| Sex | Male | 103(48.4%) |  |  |  |  |  |  |  |  |  |  |  |  |
|  | Female | 110(51.6%) | 1.105 | **0.049** | 1.000 | 1.221 | 0.998 | 0.970 | 0.908 | 1.097 | 1.124 | 0.387 | 0.863 | 1.465 |
| Ethnicity | Mestizo | 201(97.7%) |  |  |  |  |  |  |  |  |  |  |  |  |
|  | Non-Mestizo | 12(2.3%) | 1.362 | **0.003** | 1.111 | 1.670 | 0.618 | **<0.001** | 0.490 | 0.778 |  |  |  |  |
| BMI | Mean/SD | 18.6/4.3 | 0.992 | 0.171 | 0.980 | 1.003 | 1.010 | 0.085 | 0.999 | 1.021 | 1.010 | 0.482 | 0.982 | 1.040 |
|  | Median (Q1-Q3) | 17.9(15.6,20.6) |  |  |  |  |  |  |  |  |  |  |  |  |
|  | Min-Max | 9.0-38.3 |  |  |  |  |  |  |  |  |  |  |  |  |
| Length of breastfeeding | Never | 10 (4.7%) |  |  |  |  |  |  |  |  |  |  |  |  |
|  | 0-6 months | 27(12.7%) | 1.396 | **0.011** | 1.078 | 1.808 | 1.468 | **0.005** | 1.124 | 1.916 |  |  |  |  |
|  | >6 months | 174 (81.7%) | 0.874 | 0.256 | 0.694 | 1.102 | 1.368 | **0.010** | 1.077 | 1.737 |  |  |  |  |
|  | Missing | 2(0.9%) |  |  |  |  |  |  |  |  |  |  |  |  |
| FeNO (log scale) | Continuous |  | 1.176 | **<0.001** | 1.133 | 1.220 | 0.949 | **0.001** | 0.920 | 0.980 | 1.120 | **0.039** | 1.006 | 1.248 |
| **Socio-economic and household** |  |  |  |  |  |  |  |  |  |  |  |  |  |  |
| Site | Quito | 53(24.8%) |  |  |  |  |  |  |  |  |  |  |  |  |
|  | Cuenca | 98 (46.0%) | 0.365 | **<0.001** | 0.324 | 0.412 | 1.427 | **<0.001** | 1.267 | 1.607 | 1.605 | **0.027** | 1.056 | 2.439 |
|  | Portoviejo | 62 (29.1%) | 0.345 | **<0.001** | 0.301 | 0.396 | 1.117 | 0.101 | 0.979 | 1.274 | 1.489 | 0.081 | 0.952 | 2.330 |
| Maternal education level | None/Basic | 39(18.3%) |  |  |  |  |  |  |  |  |  |  |  |  |
|  | Some second/high | 146(68.6%) | 1.030 | 0.656 | 0.905 | 1.172 | 1.132 | 0.053 | 0.999 | 1.284 | 1.381 | 0.132 | 0.907 | 2.103 |
|  | Missing | 28(13.2%) |  |  |  |  |  |  |  |  |  |  |  |  |
| Dog in the house | No | 154(72.3%) |  |  |  |  |  |  |  |  |  |  |  |  |
|  | Yes | 59 (27.7%) | 0.900 | 0.065 | 0.806 | 1.007 | 1.117 | **0.036** | 1.007 | 1.240 | 0.813 | 0.209 | 0.589 | 1.123 |
| Cat in the house | No | 186 (87.3%) |  |  |  |  |  |  |  |  |  |  |  |  |
|  | Yes | 27 (12.7%) | 0.893 | 0.145 | 0.767 | 1.040 | 1.270 | **0.001** | 1.106 | 1.459 | 0.989 | 0.957 | 0.667 | 1.467 |
| Tobacco smoker in | No | 194(91.1%) |  |  |  |  |  |  |  |  |  |  |  |  |
| house | Yes | 16 (7.5%) | 1.061 | 0.527 | 0.884 | 1.274 | 1.036 | 0.690 | 0.869 | 1.236 |  |  |  |  |
|  | Missing | 3 (1.4%) |  |  |  |  |  |  |  |  |  |  |  |  |
| Mould in house | No | 183(85.9%) |  |  |  |  |  |  |  |  |  |  |  |  |
|  | Yes | 30(14.1%) | 1.150 | 0.051 | 1.000 | 1.323 | 1.181 | **0.015** | 1.033 | 1.351 | 0.568 | 0.085 | 0.298 | 1.081 |
| House location | Rural open | 47(%) |  |  |  |  |  |  |  |  |  |  |  |  |
|  | Urban/suburb | 166(%) | 1.094 | 0.151 | 0.968 | 1.236 | 1.009 | 0.878 | 0.899 | 1.132 | 0.826 | 0.196 | 0.618 | 1.104 |
| Household overcrowding | <3 per room | 154(72.3%) | 1 |  |  |  |  |  |  |  |  |  |  |  |
|  | >=3 per room | 59(27.7%) | 1.011 | 0.845 | 0.905 | 1.130 | 0.950 | 0.350 | 0.854 | 1.058 | 0.957 | 0.772 | 0.709 | 1.291 |
| House location | No | 166(%) |  |  |  |  |  |  |  |  |  |  |  |  |
| >50m main street | Yes | 98 (46.0%) | 1.100 | 0.065 | 0.994 | 1.218 | 0.980 | 0.691 | 0.890 | 1.081 | 0.949 | 0.699 | 0.726 | 1.239 |
|  | Missing | 11(5.2%) |  |  |  |  |  |  |  |  |  |  |  |  |
| Receipt of Conditional Cash Transfer | No | 185 (86.9%) |  |  |  |  |  |  |  |  |  |  |  |  |
| (bono) | Yes | 27(12.7%) | 1.539 | **<0.001** | 1.336 | 1.772 | 0.847 | **0.026** | 0.732 | 0.981 | 1.098 | 0.618 | 0.760 | 1.586 |
|  | Missing | 1 (0.5%) |  |  |  |  |  |  |  |  |  |  |  |  |
| **Personal and family history** |  |  |  |  |  |  |  |  |  |  |  |  |  |  |
| Bronchiolitis history before 2 years | No | 150(70.4%) |  |  |  |  |  |  |  |  |  |  |  |  |
|  | Yes | 57 (26.8%) | 1.008 | 0.886 | 0.902 | 1.127 | 1.333 | **<0.001** | 1.201 | 1.481 | 1.173 | 0.256 | 0.891 | 1.545 |
|  | Missing | 6 (2.8%) |  |  |  |  |  |  |  |  |  |  |  |  |
| Rhinitis last 12 m | No | 47(22.1%) |  |  |  |  |  |  |  |  |  |  |  |  |
|  | Yes | 153(71.8%) | 1.391 | **<0.001** | 1.237 | 1.564 | 1.365 | **<0.001** | 1.223 | 1.523 | 0.931 | 0.620 | 0.700 | 1.237 |
|  | Missing | 13(6.1%) |  |  |  |  |  |  |  |  |  |  |  |  |
| Skin flexion areas | No | 145(68.1%) |  |  |  |  |  |  |  |  |  |  |  |  |
|  | Yes | 55(25.8%) | 1.363 | **<0.001** | 1.221 | 1.520 | 1.020 | 0.716 | 0.916 | 1.136 | 0.971 | 0.849 | 0.719 | 1.312 |
|  | Missing | 13(6.1%) |  |  |  |  |  |  |  |  |  |  |  |  |
| Maternal asthma | No | 180 (84.5%) |  |  |  |  |  |  |  |  |  |  |  |  |
|  | Yes | 30 (14.1%) | 1.141 | 0.061 | 0.994 | 1.310 | 0.913 | 0.193 | 0.797 | 1.047 | 1.381 | **0.041** | 1.014 | 1.880 |
|  | Missing | 3 (1.4%) |  |  |  |  |  |  |  |  |  |  |  |  |
| Maternal rhinitis | No | 150 (70.4%) |  |  |  |  |  |  |  |  |  |  |  |  |
|  | Yes | 60 (28.2%) | 0.989 | 0.838 | 0.886 | 1.103 | 1.274 | **<0.001** | 1.148 | 1.413 | 0.979 | 0.887 | 0.733 | 1.309 |
|  | Missing | 3 (1.4%) |  |  |  |  |  |  |  |  |  |  |  |  |
| Paternal asthma | No | 177(%) |  |  |  |  |  |  |  |  |  |  |  |  |
|  | Yes | 25 (%) | 1.181 | **0.028** | 1.018 | 1.370 | 1.217 | **0.007** | 1.055 | 1.404 | 1.773 | **<0.001** | 1.318 | 2.387 |
|  | Missing | 11 (%) |  |  |  |  |  |  |  |  |  |  |  |  |
| Paternal rhinitis | No | 146(68.5%) |  |  |  |  |  |  |  |  |  |  |  |  |
|  | Yes | 55(25.8%) | 1.151 | **0.014** | 1.029 | 1.288 | 1.018 | 0.738 | .913 | 1.136 | 1.074 | 0.625 | .806 | 1.430 |
|  | Missing | 12(5.6%) |  |  |  |  |  |  |  |  |  |  |  |  |
| Pandemic effect (longitudinal) | Before | 7(3.3%) |  |  |  |  |  |  |  |  |  |  |  |  |
| Follow-up exceeding 16^th^ March | After | 206 (96.7%) | 1.198 | **0.012** | 1.041 | 1.380 | 0.672 | **<0.001** | 0.588 | 0.769 | 0.665 | **0.038** | 0.453 | 0.977 |

**Supplementary Table 3. Minimally adjusted (for the follow-up time) effects of the variables of the longitudinal binary outcomes indicating asthma medication uptake.** Generalized estimated equations methodology were employed to identify the potential confounders between asthma and medication uptake. FeNO – fractional exhaled nitric oxide.

| **Variable** |  | All | β2 agonist | | | | Inhaled corticosteroid | | | | Oral corticosteroid | | | |
| --- | --- | --- | --- | --- | --- | --- | --- | --- | --- | --- | --- | --- | --- | --- |
|  |  |  | OR | p-value | 95%CI - L | 95%CI - H | OR | p-value | 95%CI - L | 95%CI - H | OR | p-value | 95%CI - L | 95%CI - H |
| **Asthma characteristics** |  |  |  |  |  |  |  |  |  |  |  |  |  |  |
| Wheezing last 12 months | No | 13(6.1%) |  |  |  |  |  |  |  |  |  |  |  |  |
|  | Yes | 195 (91.6 %) | 1.300 | **0.027** | 1.030 | 1.641 | 1.167 | 0.154 | 0.944 | 1.442 | 0.799 | 0.358 | 0.496 | 1.289 |
|  | Missing | 5(2.4%) |  |  |  |  |  |  |  |  |  |  |  |  |
| Number asthma attacks | Mean/SD | 2.7/2.5 | 1.067 | **<0.001** | 1.047 | 1.088 | 1.037 | **<0.001** | 1.017 | 1.056 | 1.055 | **0.016** | 1.010 | 1.102 |
| last 12m | Median (Q1-Q3) | 2(1, 3) |  |  |  |  |  |  |  |  |  |  |  |  |
|  | Min-Max | 0-12 |  |  |  |  |  |  |  |  |  |  |  |  |
| Wheeze with exercise | No | 49(23%) |  |  |  |  |  |  |  |  |  |  |  |  |
| last 12 months (binary) | Yes | 138(64.8%) | 1.218 | **<0.001** | 1.094 | 1.355 | 1.126 | **0.021** | 1.018 | 1.245 | 0.992 | 0.952 | 0.753 | 1.306 |
|  | Missing | 26(12.2%) |  |  |  |  |  |  |  |  |  |  |  |  |
| **Medical attention history** |  |  |  |  |  |  |  |  |  |  |  |  |  |  |
| Previous doctor diagnosis asthma | No | 57(26.8%) |  |  |  |  |  |  |  |  |  |  |  |  |
|  | Yes | 146(68.5%) | 1.057 | 0.343 | 0.942 | 1.185 | 1.572 | **<0.001** | 1.401 | 1.764 | 1.263 | 0.186 | 0.893 | 1.785 |
|  | Missing | 10(4.5%) |  |  |  |  |  |  |  |  |  |  |  |  |
| Unplanned consultations last 12 m | Mean/SD | 1.9/2.4 | 1.100 | **<0.001** | 1.077 | 1.122 | 1.023 | **0.027** | 1.003 | 1.043 | 1.003 | 0.917 | 0.950 | 1.059 |
|  | Median (Q1-Q3) | 1(0-3) |  |  |  |  |  |  |  |  |  |  |  |  |
|  | Min-Max | 0-12 |  |  |  |  |  |  |  |  |  |  |  |  |
| ER last 12 months | No | 90(42.3%) |  |  |  |  |  |  |  |  |  |  |  |  |
|  | Yes | 123(57.7%) | 1.515 | **<0.001** | 1.364 | 1.683 | 1.045 | 0.368 | 0.949 | 1.151 | 0.999 | 0.992 | 0.765 | 1.304 |
| Number of ER visits last 12 months | Mean/SD | 1.9/2.8 | 1.067 | **<0.001** | 1.049 | 1.086 | 1.027 | **0.002** | 1.010 | 1.043 | 0.996 | 0.859 | 0.949 | 1.045 |
| (continuous) | Median (Q1-Q3) | 1(0, 3) |  |  |  |  |  |  |  |  |  |  |  |  |
|  | Min-Max | (0, 25) |  |  |  |  |  |  |  |  |  |  |  |  |
| Hospital/ICU attendance last 12m | No | 186(87.3%) |  |  |  |  |  |  |  |  |  |  |  |  |
|  | Yes | 27(12.7%) | 1.190 | **0.018** | 1.031 | 1.373 | 1.254 | **0.001** | 1.093 | 1.439 | 1.085 | 0.662 | 0.752 | 1.567 |
| Time since the last ER visit | Never | 176 (82.6%) |  |  |  |  |  |  |  |  |  |  |  |  |
| in the last 12 months | <30 days | 13 (6.1%) | 1.247 | **0.025** | 1.028 | 1.513 | 1.204 | 0.055 | 0.996 | 1.455 | 0.759 | 0.435 | 0.379 | 1.518 |
|  | 30-150 days | 15 (7.0%) | 0.724 | **0.004** | 0.581 | 0.902 | 2.119 | **<0.001** | 1.763 | 2.548 | 0.753 | 0.420 | 0.377 | 1.502 |
|  | >150 days | 9 (4.2%) | 1.045 | 0.717 | 0.822 | 1.329 | 1.007 | 0.953 | 0.797 | 1.273 | 1.339 | 0.247 | 0.817 | 2.192 |
| OCS >3days - composite | No | 173(81.2%) |  |  |  |  |  |  |  |  |  |  |  |  |
| Outpatient/IC/Hospital/ER | Yes | 40(18.8%) | 1.170 | **0.012** | 1.035 | 1.324 | 1.406 | **<0.001** | 1.250 | 1.581 | 1.360 | **0.036** | 1.020 | 1.813 |
| β2 agonist use over last 12m | None | 172(80.7%) |  |  |  |  |  |  |  |  |  |  |  |  |
|  | Wheezing only | 33(15.5%) | 1.317 | **<0.001** | 1.154 | 1.502 | 1.178 | 0.012 | 1.036 | 1.339 | 0.799 | 0.302 | 0.522 | 1.223 |
|  | Regularly | 8(3.8%) | 1.071 | 0.601 | 0.828 | 1.385 | 0.927 | 0.552 | 0.721 | 1.191 | 0.894 | 0.764 | 0.429 | 1.861 |
| ICS use over last 12 m | None | 188(88.3%) |  |  |  |  |  |  |  |  |  |  |  |  |
|  | Wheezing only | 7(3.3%) | 1.222 | 0.128 | 0.944 | 1.581 | 1.767 | **<0.001** | 1.381 | 2.262 | 1.290 | 0.390 | 0.722 | 2.306 |
|  | Regularly | 18(8.5%) | 0.841 | 0.066 | 0.700 | 1.012 | 1.809 | **<0.001** | 1.539 | 2.127 | 1.182 | 0.428 | 0.782 | 1.787 |

**Supplementary Table 4 Minimally adjusted (for the follow-up time) effects of the variables of the longitudinal binary outcomes indicating asthma medication uptake.** Generalized estimated equations methodology were employed to identify the potential confounders between asthma and medication intake. ICS – inhaled corticosteroids; OCS – oral corticosteroids; ER – emergency room; ICU -intensive care unit.

| Variable | Category | **HR** | **P value** | **95%-low** | **95%-high** | **HR** | **P value** | **95%-low** | **95%-high** |
| --- | --- | --- | --- | --- | --- | --- | --- | --- | --- |
| Mould in the house | No | 1 |  |  |  | 1 |  |  |  |
|  | Yes | 1.375 | 0.050 | 0.999 | 1.891 | 1.368 | 0.055 | 0.993 | 1.885 |
| Number of medical attentions | Count | 1.051 | 0.048 | 1.000 | 1.104 | 1.054 | 0.031 | 1.005 | 1.106 |
| Pandemic effect | Before | 1 |  |  |  | 1 |  |  |  |
|  | After | 0.366 | <0.001 | 0.233 | 0.574 | 0.361 | <0.001 | 0.230 | 0.567 |
| ICS intake a month before attack | No | 1 |  |  |  | 1 |  |  |  |
| (time-varying) | Yes | 0.680 | 0.045 | 0.466 | 0.992 | 0.675 | 0.038 | 0.465 | 0.979 |
| Physician’s diagnosis | No | 1 |  |  |  | 1 |  |  |  |
|  | Yes | 1.128 | 0.585 | 0.733 | 1.735 | 1.060 | 0.802 | 0.674 | 1.666 |
| B2 agonist intake a month before attack | No | 1 |  |  |  | 1 |  |  |  |
| (time-varying) | Yes | 1.299 | 0.424 | 0.684 | 2.465 | 1.108 | 0.755 | 0.582 | 2.111 |
| Physician’s diagnosis × β2 agonist |  | 1 |  |  |  | 1 |  |  |  |
| (interaction) |  | 1.830 | 0.088 | 0.914 | 3.664 | 2.195 | 0.028 | 1.091 | 4.419 |

**Supplementary Table 5. Sensitivity analyses to the missing observations for the paternal asthma for the most parsimonious model.** We have considered 2 different extreme scenarios described: Scenario 1: missing physician’s diagnosis set to 0 (diagnosis set to no asthma). Scenario 2: missing physician’s diagnosis set to 1 (diagnosis set to asthma). The magnitude and the strength of the adjusted associations remain relatively robust across these two extreme scenarios. Participants with missing information for doctor diagnosis were less likely to have had a recurrence than those with this information, although the evidence is not strong (P=0.04).
